# Bats and Viruses: Current Research and Future Trends 

**DOI:** 10.3201/eid2703.204561

**Published:** 2021-03

**Authors:** Eugenia Corrales-Aguilar, Martin Schwemmle, editors, David Hewitt

**Affiliations:** US Department of Housing and Urban Development, Washington, DC, USA

**Keywords:** zooonoses, virology, mammals, bats, viruses

Fluttering above us at dusk, bats evoke awe and wonder as they signal the coming of the night. Bats are native to every continent but Antarctica, and they often live in or enter human-occupied spaces. They are highly diverse, both phylogenetically and ecologically. Bats also carry zoonotic diseases, including many of the viruses discussed in the new book *Bats and Viruses*, edited by Eugenia Corrales-Aguilar and Martin Schwemmle (Figure). Bats’ diversity, frequent proximity to humans, and carriage of zoonotics make them an important and often poorly understood component of public health and infectious disease.

**Figure:**
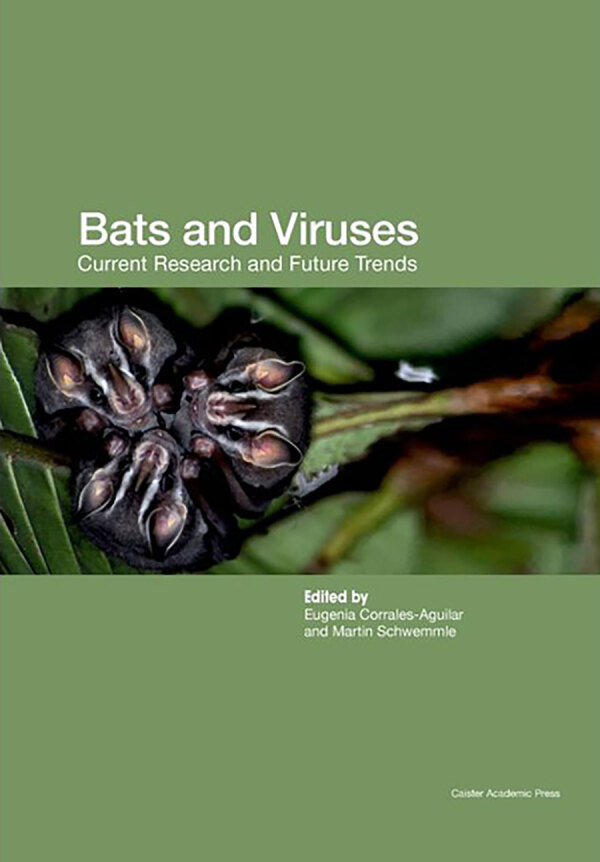
Bats and Viruses: Current Research and Future Trends

*Bats and Viruses *sets out to review hot topics in current research, and it also provides background information accessible to those without specific expertise in this particular field. It is a highly technical volume that is unlikely to be readily accessible to a general audience. It is, however, understandable to a reader outside of the immediate technical area of bats and viruses, especially those with technical backgrounds in zoonotics and infectious disease; wildlife biology, including wildlife ecology; evolutionary biology, and phylogenetics; and molecular and cellular biology.

In addition to a review of current topics, the book provides background in bat biology and virology; this is of great value to a reader who is not deeply experienced in this technical area. Each chapter includes an extensive references section, providing even more opportunity to delve deeply into the background.

The organization of the book is logical. About half of the chapters are organized by taxonomy of the viruses being covered: flaviviruses, alphaviruses, influenza A–like viruses, coronaviruses, hantaviruses, polyomaviruses. Two chapters cover immunity in bats: one on innate immunity, the other on adaptive immunity. Three chapters address techniques used to study virology of bats: isolation of viruses; in vivo techniques, including coverage of bat husbandry; metagenomics. 

Given the current pandemic, the chapter that many will open up to first is “Bats and Coronaviruses,” by Susanna K. P. Lau, et al. This chapter is clearly understandable to a reader without expertise in the field of bat biology and virology, contains a lucid history of coronaviruses, and focuses in on some of the more well-known coronaviruses, such as severe acute respiratory syndrome and Middle East respiratory syndrome viruses. Although other chapters cover coronaviruses, this information is not indicated in the index; a more comprehensive index would be helpful to guide the reader across the multiplicity of topics covered in this multiauthored volume.

This book would be useful for scientific libraries seeking a technical overview of current topics in bats and viruses with an emphasis on human health effects. It is accessible to readers with backgrounds in infectious disease, wildlife biology, and molecular or cellular biology and would be of interest to those readers who want to know more about how those different fields interact.

